# Plasma extracellular vesicle: a novel biomarker for neurodegenerative disease diagnosis

**DOI:** 10.20517/evcna.2024.56

**Published:** 2024-09-30

**Authors:** Xinrui Zhao, Shenglin Huang

**Affiliations:** Department of Integrative Oncology, Fudan University Shanghai Cancer Center, Shanghai Key Laboratory of Medical Epigenetics, Institutes of Biomedical Sciences, Fudan University, Shanghai 200032, China.

**Keywords:** Neurodegenerative diseases, plasma extracellular vesicle, Tau, TDP-43, diagnostic marker

## Abstract

Extracellular vesicles (EVs) are membrane-bound structures that carry proteins, lipids, RNA, and DNA, playing key roles in cell communication and material transport. Recent research highlights their potential as disease biomarkers due to their stability in bodily fluids. This study explores using tau and TDP-43 proteins in plasma EVs as diagnostic biomarkers for frontotemporal dementia (FTD) and amyotrophic lateral sclerosis (ALS). Analyzing plasma EVs from clinical cohorts, the study found that the 3R/4R tau ratio and TDP-43 levels effectively differentiate between diagnostic groups with high accuracy. Notably, plasma EV biomarkers demonstrate higher diagnostic accuracy and stability compared to direct plasma testing, providing new insights and approaches for future research and clinical practice. Further research is needed to validate these biomarkers in diverse populations and to establish standardized protocols. Future studies should continue to explore the potential of EV biomarkers in a broader range of neurodegenerative diseases and delve deeper into the mechanisms of EV secretion and sorting to enhance their diagnostic utility.

## MAIN TEXT

Extracellular vesicles (EVs) are small membrane-bound structures secreted by cells, containing proteins, lipids, RNA, and DNA. They play crucial roles in cell-cell communication, material transport, and waste removal^[1,2]^. In recent years, EVs have garnered significant attention as disease biomarkers. EVs protect their contents from enzymatic degradation, making them stable carriers of biomarkers in bodily fluids such as blood. A recent study published in Nature Medicine identified the potential of tau and TDP-43 proteins in plasma EVs as a diagnostic biomarker for frontotemporal dementia (FTD) and amyotrophic lateral sclerosis (ALS)^[3]^.

**Figure 1:**
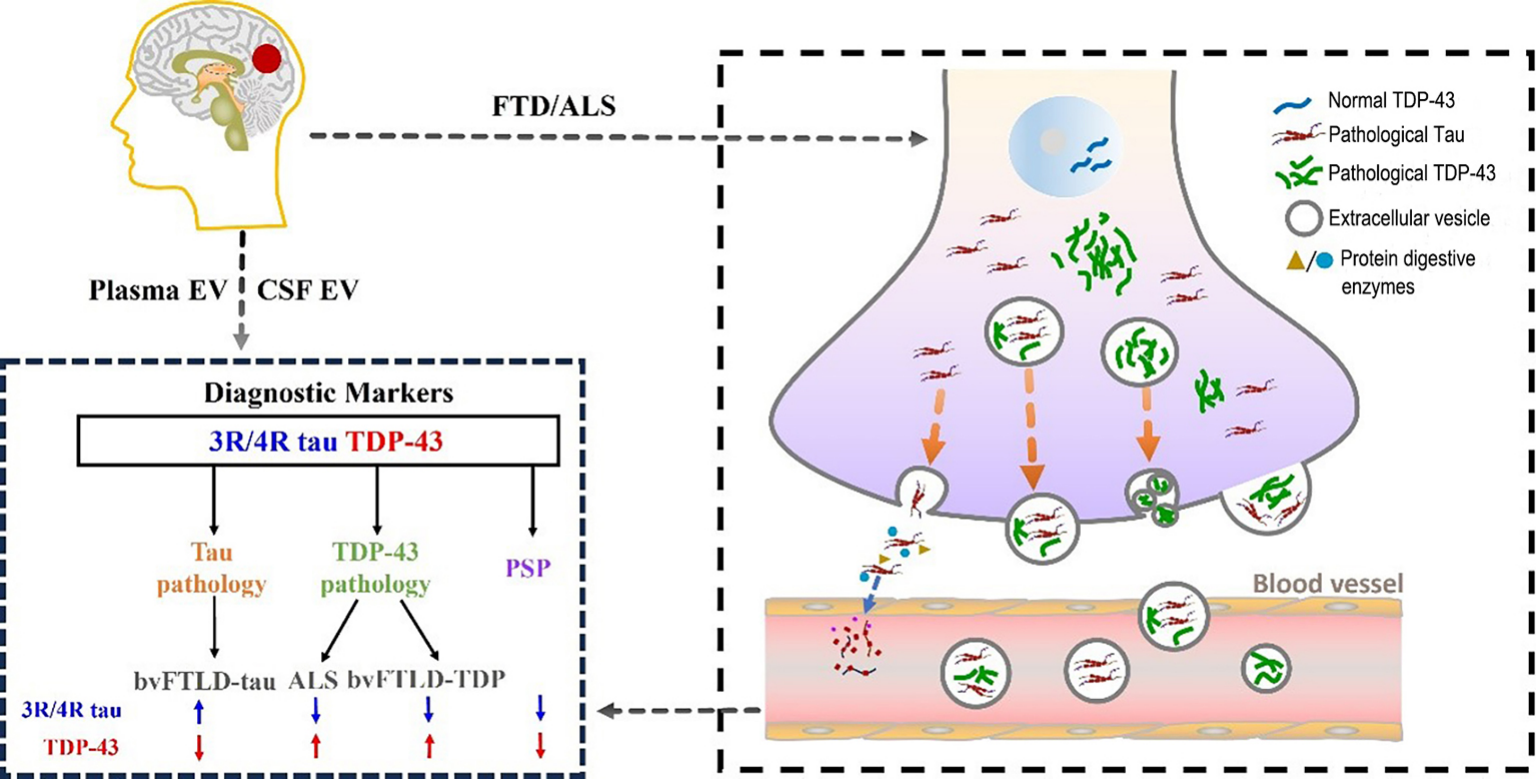
Plasma extracellular vesicle tau and TDP-43 proteins aid in the diagnosis of FTD and ALS spectrum disorders. In neurons of patients with FTD and ALS spectrum disorders, alternative splicing of the tau gene produces 3R tau and 4R tau isoforms. Under normal conditions, 3-repeat and 4-repeat tau are present in approximately equal amounts in the adult brain. However, in patients with FTD and ALS, the ratio of 3R to 4R tau may become abnormal. Some tau protein is directly secreted into the extracellular space and is cleaved by extracellular proteases, resulting in fragmented tau in the extracellular fluid and very low levels of full-length tau protein. Another portion of tau protein is released into the extracellular environment in the form of EVs. Additionally, in FTD and ALS patients, TDP-43 protein abnormally accumulates in the cytoplasm, forming inclusions (abnormal nuclear localization), with some being carried extracellularly via EVs. By collecting EVs from patient blood and cerebrospinal fluid, TDP-43 levels and 3R/4R tau ratios can be measured. Based on the detection thresholds, different pathologies and diagnostic groups can be distinguished. The blue arrow represents downregulation, and the red arrow represents upregulation. FTD: Frontotemporal dementia; ALS: amyotrophic lateral sclerosis; Evs: extracellular vesicles; CSF: cerebrospinal fluid; PSP: progressive supranuclear palsy.

This study aims to explore the feasibility of using tau and TDP-43 proteins in plasma EVs as diagnostic biomarkers for FTD and ALS [Figure 1]. FTD encompasses a group of neurodegenerative diseases involving the frontal and temporal lobes, including behavioral variant FTD (bvFTD), semantic variant primary progressive aphasia (svPPA), and non-fluent variant primary progressive aphasia (nfvPPA)^[4]^. These diseases, along with ALS, form a continuum with overlapping symptoms, genetics, and molecular pathology. The abnormal cytoplasmic aggregation of TDP-43 and the accumulation of tau proteins are key pathological features of FTD and ALS^[5]^. However, the lack of specific biomarkers has limited the early diagnosis and monitoring of treatment efficacy for these diseases, which currently rely on invasive tests and genetic analyses.

TDP-43 is a nuclear protein that primarily functions in the cell nucleus. However, abnormal cytoplasmic aggregation of TDP-43 is commonly observed in neurodegenerative diseases. The main function of tau proteins is to stabilize microtubules in neurons. Their microtubule-binding domains contain either three (3R tau) or four (4R tau) repeat sequences, which are generated by alternative splicing of the tau gene. Normally, 3-repeat and 4-repeat tau are present in approximately equal amounts in the adult brain. However, in certain neurodegenerative diseases (e.g., FTD and ALS), the ratio of 3R to 4R tau can become abnormally altered, with both isoforms potentially accumulating and aggregating abnormally. Given that tau proteins in extracellular fluids are often fragmented due to protease activity in the extracellular environment, and that EVs protect proteins from degradation, the researchers focused on EVs as a potential source of diagnostic biomarkers. The researchers centrifuged the collected cerebrospinal fluid (CSF) and plasma at 10,000 g to obtain the pellet containing medium-sized extracellular vesicles (mEVs). Subsequently, size exclusion chromatography (SEC) was applied to the supernatant to isolate small extracellular vesicles (sEVs). Immunoprecipitation mass spectrometry (IP-MS) analysis revealed the presence of full-length tau in the EVs from both CSF and plasma, allowing differentiation between the 3-repeat and 4-repeat isoforms. The concentrations of 3-repeat and 4-repeat tau isoforms in plasma EVs were quantified using a sandwich immunoassay. Therefore, the authors aimed to investigate whether TDP-43 and the 3R/4R tau ratio in EVs could serve as diagnostic biomarkers for FTD and ALS, providing a more convenient and rapid diagnosis for these diseases.

To address these challenges, this study systematically measured the proportions of 3-repeat and 4-repeat tau isoforms and the levels of TDP-43 protein in plasma EVs by analyzing samples from two large clinical cohorts. The study included several diagnostic groups: ALS characterized by TDP-43 proteinopathy, progressive supranuclear palsy (PSP) characterized by 4R tau-dominant tauopathy, and bvFTD patients with either tau or TDP-43 pathology (classified as FTLD-tau and FTLD-TDP groups, respectively), along with healthy controls. The results showed that the 3R/4R tau ratio was significantly lower in PSP patients compared to other groups, while bvFTD patients exhibited a significantly higher ratio. ALS patients and bvFTD patients with TDP-43 pathology had significantly elevated levels of EV TDP-43. These biomarkers were closely correlated with the severity of clinical and neuropsychological indicators, achieving diagnostic accuracies of over 0.9.

Specifically, the study involved isolating and analyzing medium-sized vesicles and small vesicles from plasma, using immunoprecipitation-mass spectrometry (IP-MS) to identify full-length tau proteins in EVs, thereby distinguishing 3-repeat and 4-repeat tau isoforms. Furthermore, sandwich immunoassays were employed to quantify the concentrations of 3-repeat and 4-repeat tau isoforms in plasma EVs, allowing the measurement of their ratios. EVs transport pathological tau and TDP-43 between cells, and the detection of TDP-43 in EVs likely indicates its pathological relocation from the nucleus to the cytoplasm, as nuclear export is a prerequisite for TDP-43 incorporation into EVs. To confirm the neuronal origin of EVs, the researchers used immunomagnetic beads to isolate L1 cell adhesion molecule (L1CAM)-positive EVs, an important neural cell adhesion molecule, showing that the majority of plasma EV tau and EV TDP-43 were present in L1CAM EVs^[6]^. Although there is still some debate regarding whether L1CAM can serve as a marker for neuron EVs^[7]^, a recent study has suggested that L1CAM can be considered a reliable biomarker for neuron-derived EVs, providing some evidence to support its use as a marker for neuron EVs^[6]^.

The results were consistent across the initial cohort (DESCRIBE) and the validation cohort (Sant Pau). The DESCRIBE cohort established threshold values (sEV 3R/4R tau ratio: 0.77 and 1.27; sEV TDP-43: 17.87 pg/mL and 56.18 pg/mL) to differentiate between diagnostic groups through mixed modeling, and the Sant Pau cohort yielded similar thresholds (sEV 3R/4R tau ratio: 0.78 and 1.28; sEV TDP-43: 17.85 pg/mL and 57.34 pg/mL), and potential confounders (age, sex and disease duration) showed no influence on plasma biomarker levels, further validating the reliability and applicability of these findings. The integration of TDP-43 levels with EV 3R/4R tau ratios in EVs may aid in the biomarker-based diagnosis of ALS, FTD, and FTD spectrum disorders.

The findings suggest that measuring tau and TDP-43 levels in plasma EVs could serve as an effective, non-invasive molecular diagnostic tool for FTD and ALS. These biomarkers not only aid in early diagnosis but also monitor disease progression and treatment efficacy. Additionally, this study offers new insights and approaches for future research and clinical practice in neurodegenerative diseases, especially in achieving personalized medicine and precise diagnosis. By establishing standardized sample collection and analysis protocols, these biomarkers could be widely applied in clinical practice, further advancing the research and treatment of FTD and ALS.

Despite the promising potential of plasma EV tau and TDP-43 levels as diagnostic biomarkers for FTD and ALS, several limitations need further investigation and validation. First, these findings require verification in more diverse and larger populations to ensure their generalizability and reliability. Second, standardized operating procedures (SOPs) for sample collection and analysis need to be established, including clear cutoff values to ensure comparability and reproducibility across different laboratories. Additionally, employing more sensitive detection methods, such as nucleic acid-linked immunosorbent assay (NULISA), could improve detection sensitivity and accuracy.

This study raises several important discussion points. First, plasma EV biomarkers demonstrate higher diagnostic accuracy and stability compared to direct plasma testing, primarily because EVs protect their contents from degradation. This advantage makes EV biomarkers promising candidates for diagnosing and monitoring other neurological diseases, such as Alzheimer’s and Parkinson’s disease. Second, further research into the mechanisms of EV biomarker secretion and sorting will help elucidate their roles in disease processes and potentially reveal new therapeutic targets. Moreover, the nucleic acids in EVs, particularly RNA, warrant further investigation as they are enriched in the nervous system (e.g., circular RNA) and exhibit alternative splicing^[8]^. These characteristics may provide additional diagnostic information and insights into disease mechanisms.

Future research should continue to explore the potential of EV biomarkers in a broader range of neurodegenerative diseases and delve deeper into the mechanisms of EV secretion and sorting under disease conditions. This will not only enhance current diagnostic methods but also provide crucial scientific evidence for developing new therapeutic strategies. Through these efforts, we hope to achieve earlier and more accurate diagnoses of neurodegenerative diseases, ultimately improving patient outcomes and quality of life.

## DECLARATIONS

### Copyright

© The Author(s) 2024.
